# What Happens in a 5-Year Follow-Up of Benign Thyroid Nodules

**DOI:** 10.1155/2014/459791

**Published:** 2014-11-04

**Authors:** Roberto Negro

**Affiliations:** Division of Endocrinology, “V. Fazzi” Hospital, Piazza F. Muratore, 73100 Lecce, Italy

## Abstract

To determine an optimal time for follow-up of benign thyroid nodules, we retrospectively evaluated 249 euthyroid patients with uni-multinodular goiter, who underwent annual visit, and significant events that occurred in 5 years' time were registered. A significant event (appearance of new nodule, increase of nodule diameter >50%, appearance of compressive symptoms, thyroidectomy, repetition of FNA on the same nodule, and execution of FNA on new nodule) occurred in 26.1% of patients, with more than one event occurring in the same patient in 27.7% of cases. The majority of events (71.9%) were observed at 24- and 36-month follow-up visit. These results suggest that a patient diagnosed with benign nodular goiter may be safely followed-up at a 2-3-year interval time.

## 1. Introduction

The prevalence of palpable thyroid nodules has been estimated to be around 3–7%, while the prevalence on nonpalpable nodules, detected by thyroid ultrasound or incidentally discovered by MRI or carotid Doppler ultrasound, is much higher (20–76%) [1–3]. Moreover, 20–48% of patients with a palpable nodule have another nodule detected by ultrasound [4, 5]. Once a thyroid nodule has been detected, further investigations are required, like fine-needle aspiration (FNA), thyroid function test, or scintigraphy [6]. Once the diagnosis has been made, the patients may be referred to surgery, radioiodine therapy, laser/radiofrequency ablation, or follow-up. In patients residing in iodine deficient areas, a medical treatment with levothyroxine may be advised. In AACE-AME-ETA guidelines for thyroid nodules, it is indicated that the optimal time interval for patients follow-up is 6–18 months, in lack of specific studies that address this issue [6].

Given the high prevalence of thyroid nodules and the number of patients with cytologically benign nodules with a normal thyroid function that periodically should be checked by ultrasound and thyroid function tests, it is easy to understand the global impact on the health care system deriving from such disease.

The aim of this study is to ascertain an optimal time interval of consultations, in order to guarantee an effective monitoring, but avoid an excessive and useless execution of consultations, function test, and ultrasound, for a benign disease that is also often asymptomatic.

## 2. Methods

We retrospectively selected patients who in 2007-2008 had their first visit for thyroid disease and had been diagnosed with euthyroid unimultinodular goiter and had cytological benign exam and completed their follow-up for five years (2012-2013), with at least 3 visits during this period (baseline, 60 months, and at least one other visit in between). As per our institutional protocol, patients referred to our clinic for thyroid nodule undergo ultrasound and TSH. Patients are submitted to FNA in case of nodules greater than 10 mm, irrespective to presence or absence of characteristics of malignancy and in case of nodule smaller than 10 mm, just in presence of characteristics of malignancy. Then, from the initial population of thyroid patients we excluded those with hypothyroidism, hyperthyroidism, drugs interfering with thyroid function (as listed in the ATA/AACE guidelines), and positive thyroid antibodies (when available); we also excluded those patients who, after their initial workup, underwent thyroid surgery, radioiodine therapy, or ethanol ablation, were lost at follow-up, had nodules smaller than 5 mm, and were submitted to suppressive therapy with levothyroxine.

From such selected population, the following data were recorded: sex, age, TSH, number of nodules, diameter of the nodule in case of uninodular goiter, and diameter of the major nodule in case of multinodular goiter; for the aim of the study, we considered in five-year follow-up the following events as relevant: appearance of new nodules larger than 10 mm; appearance of new nodules smaller than 10 mm with characteristics of malignancy; increase in nodule diameter greater than 50% from baseline; appearance of dyspnea, dysphonia, dysphagia, and local discomfort; execution of a FNA on the same nodule already examined at baseline or on a new nodule; any other situation that indicates surgery or any other treatment that excluded the patient from follow-up; development of thyroid dysfunction.

Ultrasounds were performed with a 7.5 MHz linear probe (Logiq 7 GE or MyLab25 Gold Esaote) by three endocrinologists with consolidated experience in thyroid ultrasound; the three diameters (anteroposterior, transverse, and longitudinal) of each nodule were usually reported; the major of the three diameters was always reported and used for the present study.

Thyroid cytology categories followed the British Thyroid Association system (THY1–5).

Permission for this review and analysis was granted by the Investigational Review Board at the “V. Fazzi” Hospital. Results are presented according to patient and nodule, respectively, and compared using a *t*-test or a *χ* ^2^ test, as appropriate. Values of *P* < 0.05 are considered significant.

## 3. Results

In 2007-2008, 1.772 patients had their first visit for thyroid disease. Of these, 555 were hypothyroid, 98 were hyperthyroid and treated with antithyroid drugs, 75 underwent radioiodine treatment for Graves' disease or Plummer adenoma, 100 underwent thyroid surgery for compressive symptoms or malignancy, 527 had nodules smaller than 5 mm, were lost at follow-up, or had less than 3 visits, 100 had chronic autoimmune thyroiditis in euthyroidism, 25 were treated with ethanol ablation for cystic nodules, and 43 underwent suppressive therapy with levothyroxine for nodular goiter. This latter group of patients spontaneously chose to be treated with levothyroxine, after exhaustive explanation; the treatment was not assigned on medical decision basis; then, the exclusion of this group should not represent a bias in analysis of results. Then, the study population consisted of 249 patients with euthyroid uni-/multinodular goiter, with benign FNA.

Characteristics of study population are described in Table 1.

Almost all patients had 5 consultations (4.98 ± 1.0). A total of 82 events occurred in 65/249 patients (26.1%) in 5-year follow-up period. The events were the following: 38 new nodules, 23 increase of nodule diameter >50% in respect to baseline, 5 increase of nodule diameter <50% but appearance of local discomfort with compressive symptoms, 6 thyroidectomies (with 2 having incidentally discovered micropapillary cancer at histological exam), 5 repetitions of FNA on the same nodule detected at baseline, and 5 FNA on new nodules. The reaspiration of the 5 nodules was done in those who had an increase of diameter >50% in respect to baseline and diameter >20 mm.

In 18/65 patients we recorded more than one event (e.g., increase of nodule diameter >50% and FNA). No patients developed thyroid dysfunction, nor was the appearance of metastatic lymph nodes observed. Of the 5 nodules that were reaspirated, no false-negative FNA was detected. None had a spontaneous reduction of thyroid nodule greater than 50% in respect to baseline.

**Figure 1:**
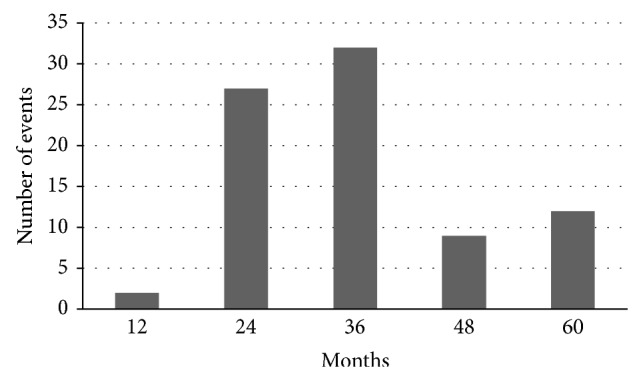
Distribution of events during follow-up.

The 82 events occurred at 36.3 ± 12.8 months from baseline. 71.9% of events (59/82) were detected at 24- and 36-month follow-up visits (*P* < 0.01); appearance of new nodules and increase of nodule >50% represent 50% of all events that occurred in 5-year follow-up (*P* < 0.01) (Figure 1).

## 4. Discussion

Given the high prevalence of benign nodular disease in the general population, an optimal management of such disease would be useful in order to adequately monitor the patients, while minimizing useless consultations, thyroid function test, and ultrasounds. Data of the present study show that, of all the possible events considered, 72% were detected between 2 and 3 years after the initial evaluation and that it is almost unnecessary to reevaluate the patients in 12 months. Indeed, this is the first study where in a 5-year follow-up patients were strictly and regularly followed up in this time period and, differently from other studies, all the possible events that may occur in the natural history and management of a thyroid nodule were considered.

Several retrospective studies investigated the natural course of benign thyroid nodules and the possible usefulness of a reaspiration, trying to help the clinician in managing these patients. In a 5-year follow-up study, Brander et al. found that, of 34 benign individual nodules, about one-third had grown and one-fourth had diminished or disappeared and, in 12% of cases, new nodules were detected; of those who underwent another FNA, no false-negative case was diagnosed [7]. In a Japanese study, Kuma et al. reexamined 134 patients 10 years after the first assessment; results showed that in a considerable number of cases nodules shrank or disappeared and in about 20% they enlarged, and all but one were confirmed to be benign at cytological exam [8]. Another investigation from Germany that collected either functioning or nonfunctioning benign nodules showed that half of the nodules increase by 30% at 3 years [9]. An increase by at least 15% was detected in 89% of nodules after a 5-year follow-up and, of those who were reaspirated for significant increase of volume, just 1/74 was revealed to be malignant. Data from the same study demonstrated that nodules with greater cystic content were less likely to grow than solid nodules. The abovementioned studies are hardly comparable because of differences in follow-up time, criteria used to define nodule enlargement, other than ethnic differences, or environmental factors like daily iodine intake; but taking data together, one can conclude that the natural course of benign thyroid nodules is characterized by a slow and progressive growth, and another common finding is that false-negative results have been found to be quite rare in all the records. A debated issue in this field involves the usefulness of reaspirating a nodule that has been classified as benign at the first evaluation. In a study by Lucas et al. published 20 years ago, 116 female patients underwent repetition of FNA after 1 year of the first FNA [10]. The authors detected no changes in the size or consistency of patients' nodular goiters, and no false-negative case was revealed. Few years later, a series composed of 45 patients who underwent one or two FNA after the first benign diagnosis showed no false-negative result, and all the seven patients who underwent thyroidectomy for compressive symptoms revealed a benign histological exam [11]. Then, in terms of follow-up for patients with a diagnosed benign thyroid nodule, a pivotal point to be addressed is when and in which cases the repetition of FNA may be indicated. A study by Rosário and Purisch consisting of 895 nodules put in evidence that the repetition of FNA is useful in detecting false-negative cases, when the nodule shows characteristics of malignancy at ultrasound rather than when it grows [12]. This concept was more recently confirmed by a multicentre study stating that evaluating 700 nodules initially diagnosed as benign showed that false-negative results were found in 1–3% of cases (depending on the centre) and that the likelihood of a cytologically benign nodule with suspicious US characteristics being malignant was higher than that of one without suspicious US (4.7% versus 0.8%; *P* < 0.05) [13]. The same low rate of false-negative FNA was demonstrated by the studies by Torre and Oertel, while we have to be reminded that, in about 2/3 of cases, false-negative results pertain to nodules larger than 30 mm [14–16]. Finally, a more recent study by Nou et al. suggests that because thyroid malignancies appear adequately treated despite detection at a mean of 4.5 years after initial falsely benign cytology, the authors suggest repeating thyroid nodule evaluation 2–4 years after the first FNA [17]. Although the selection of patients and the outcome of our study and the study by Nou et al. are different, our data allow reaching similar conclusions, suggesting a long term follow-up.

In clinical practice, the interval time of 2-3 years suggested by the abovementioned results should anyway take into account the US characteristics of nodules. In the evaluation of data, we did not consider the nodule's features like echogenicity, shape, margins, or vascularity, which are of pivotal importance when planning patient's follow-up.

## 5. Conclusions

In conclusion, in a population of patients with uni-/multinodular goiter and benign FNA, about 1/4 develops a further event after initial work-out in 5-year follow-up. In half of the cases, appearance of a new nodule or an increase by more than 50% of the initial nodule's diameter occurs. The vast majority of new events were observed at 24- and 36-month follow-up visits

These results confirm that a patient diagnosed with benign nodular goiter may be safely followed up at 2-3-year interval time. This lapse of time seems to be longer than the 6–18 months suggested by current guidelines and, if routinely implemented, would hesitate in dramatic savings for the health care system, without entailing additional risks for the patient's health.

## Figures and Tables

**Table 1:** Characteristics of study population.

	Baseline	12 mo	24 mo	36 mo	48 mo	60 mo	*P*
Number of patients	249	249	247	245	245	243	
F/M	209/40	209/40	207/40	205/40	205/40	203/40	<0.01
Age (yr)	54.7 ± 12.7						
TSH (mIU/L)	1.5 ± 0.9	1.5 ± 0.9	1.5 ± 1.5	1.4 ± 0.7	1.5 ± 0.9	1.5 ± 0.9	ns
UNG/MNG	113/136	113/136	113/134	113/132	113/132	113/130	ns
Number of nodules	2 ± 1.2	2 ± 1.3	2.1 ± 1.3	2.1 ± 1.3	2.3 ± 1.3	2.1 ± 1.2	ns
Diameter of major nodule (mm)	15.6 ± 6.4	16.3 ± 6.9	16.8 ± 6.4	16.4 ± 7.0	17 ± 7.5	16.7 ± 7.4	ns
